# Non‐stoichiometric Sodium Chloride Reprograms Ionic Homeostasis to Enhance Antitumor Immunity

**DOI:** 10.1002/mco2.70534

**Published:** 2025-11-29

**Authors:** Chongxiao Wang, Yuan Deng, Heng Liang, Arabella H. Wan, Shijia Yan, Min Xiao, Chuwei Liu, Juan Fang, Zhi Wang, Guohui Wan

**Affiliations:** ^1^ State Key Laboratory of Anti‐Infective Drug Discovery and Development, National‐Local Joint Engineering Laboratory of Druggability and New Drug Evaluation, Schools of Pharmaceutical Sciences & Materials Science and Engineering Sun Yat‐Sen University Guangzhou China; ^2^ Hospital of Stomatology, Guanghua School of Stomatology Sun Yat‐Sen University Guangzhou China

1

Dear Editor,

The tumor microenvironment (TME) exhibits profound ionic dysregulation, with accumulation of extracellular potassium (K⁺) emerging as a potent immunosuppressive factor that impairs T cell metabolism, cytokine secretion, and effector differentiation [1]. Elevated K⁺, often released from dying tumor cells, creates a hostile ionic niche that inhibits dendritic cell (DC) activation and CD8⁺ T cell cytotoxicity [1, 2]. While strategies such as ion‐channel blockade and transition metal‐based nanomaterials have been proposed to rebalance ionic homeostasis [3, 4], these approaches suffer from limited biocompatibility, systemic toxicity, and complexity in formulation. Therefore, a simple, safe, and scalable method for modulating ionic conditions within tumors remains an unmet need. In this study, we present non‐stoichiometric sodium chloride (n‐NaCl), particularly Cl^−^‐excess n‐NaCl, as a clinically translatable ionic modulator that selectively depletes intracellular K⁺ in immune cells, reprograms DC function, and augments CD8⁺ T cell‐mediated tumor clearance.

To generate non‐stoichiometric NaCl formulations, we established a low‐voltage electrodialysis system equipped with cation‐ and anion‐selective membranes. This setup allowed the production of Na⁺‐rich and Cl^−^‐rich saline solutions through asymmetric ion migration under sub‐electrolytic conditions, resulting in approximately 30% deviation from the standard 1:1 Na⁺/Cl^−^ ratio (Figure 1A). Both formulations maintained isotonicity and neutral pH, ensuring compatibility with cellular systems. Structural analysis by atomic force microscopy (AFM) and electron paramagnetic resonance (EPR) revealed distinct surface morphologies and magnetic environments for Cl^−^‐excess versus standard NaCl, confirming the altered ionic structure and solvation states of n‐NaCl formulations (Figure 1A).

**FIGURE 1:**
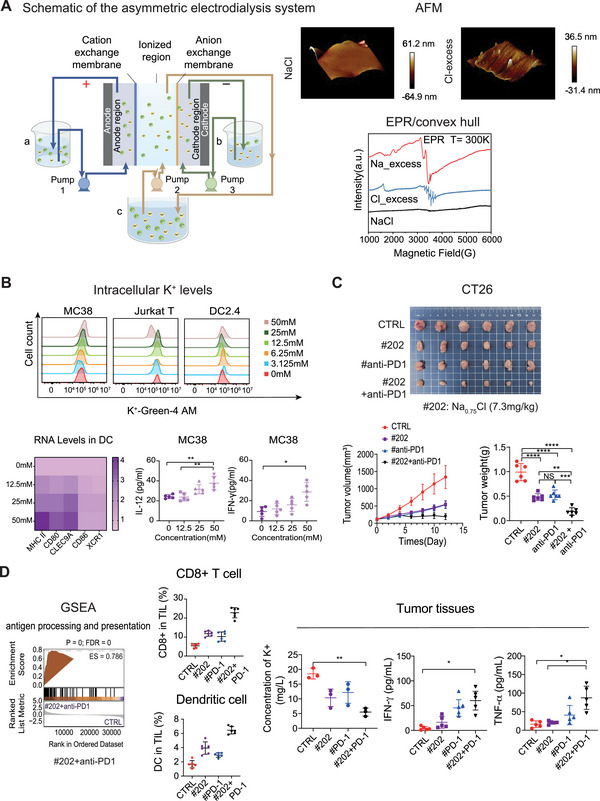
Non‐stoichiometric NaCl selectively depletes intracellular K⁺ and enhances antitumor immunity. (A) Generation and characterization of non‐stoichiometric NaCl with altered ionic states. (B) Selective K⁺ depletion reprograms dendritic cells and enhances cytokine production. (C) Cl^−^‐excess n‐NaCl inhibits CT26 tumor growth and synergizes with PD‐1 blockade in vivo. (D) Ionic modulation enhances immune priming and intratumoral cytokine levels. anti‐PD1, anti‐programmed cell death‐1 antibody; a.u., arbitrary units; AFM, atomic force microscopy; CTRL, control; DC2.4/DC, dendritic cells; EPR, electron paramagnetic resonance; ES, enrichment score; GSEA, gene set enrichment analysis; IFN‐γ, interferon‐gamma; IL‐12, interleukin‐12; Jurkat T, Jurkat T lymphocyte cell line; K⁺‐Green‐4 AM, potassium‐sensitive fluorescent dye; MC38 and CT26, murine colon carcinoma cell lines; TIL, tumor‐infiltrating lymphocyte; TNF‐α, tumor‐necrosis factor alpha.

Cl^−^‐excess n‐NaCl selectively depleted intracellular K⁺ in a dose‐dependent manner in Jurkat T and DC2.4 dendritic cells, while paradoxically increasing K⁺ levels in MC38 tumor cells (Figure 1B, upper panel). This cell‐type selectivity likely reflects differences in membrane potential, cytoplasmic buffering, and transporter abundance. Immune cells rely on rapid ionic fluxes to trigger activation, rendering them more vulnerable to Cl^−^‐driven shifts, whereas tumor cells upregulate Na⁺/K⁺‐ATPase and cation‐chloride cotransporters (NKCC1/KCC) to sustain proliferative homeostasis [2]. This mechanistic divergence highlights a therapeutic window in which ionic modulation enhances immune cell function without directly harming tumor cells. Such selective ionic modulation is particularly advantageous in the TME, where nonspecific ionic perturbation often risks harming immune cells. In dendritic cells, reduced intracellular K⁺ induced robust phenotypic and transcriptional maturation, as shown by increased expression of MHC‐II and co‐stimulatory genes (Figure 1B, lower left heatmap). Functionally, these changes led to elevated IL‐12 and IFN‐γ production in co‐culture supernatants and enhanced cytotoxic activity of CD8⁺ T cells [1, 4], as evidenced by increased tumor cell death in MC38 co‐culture systems (Figure 1B, lower right). These observations suggest that Cl^−^‐excess n‐NaCl reprograms DC function through ionic signaling, enhancing their capacity to bridge innate and adaptive immunity within the tumor milieu.

To evaluate in vivo efficacy, we administered Cl^−^‐excess n‐NaCl intratumorally in CT26 tumor‐bearing mice, and observed significantly reduced tumor burden compared to control and anti‐PD‐1 monotherapy (Figure 1C). Notably, the Cl^−^‐excess formulation exhibited a superior antitumor effect, suggesting that its immunomodulatory properties translated effectively into therapeutic outcomes. Tumor inhibition rates (TIR) calculated from final tumor weights were ∼42% for Cl^−^‐excess monotherapy, ∼28% for anti–PD‐1 in CT26, whereas the combination achieved ∼63%, highlighting synergistic benefit.

Mechanistically, tumors treated with Cl^−^‐excess n‐NaCl showed transcriptional activation of antigen presentation and immune response pathways, as revealed by gene set enrichment analysis (Figure 1D, left). Flow cytometry showed increased infiltration of CD8⁺ T cells and CD11c⁺ dendritic cells (Figure 1D, middle). Ion quantification assays confirmed reduced intratumoral K⁺, while ELISA demonstrated elevated IFN‐γ and TNF‐α protein levels (Figure 1D, right), reinforcing the link between ionic reprogramming and enhanced immunogenicity in situ. Notably, we did not assess Treg or MDSC populations in this study, which should be addressed in future work.

Collectively, these findings highlight the promise of non‐stoichiometric NaCl formulations as readily translatable ionic modulators. Unlike synthetic nanomaterials or chemical inhibitors that often face challenges in clinical deployment due to toxicity, instability, or manufacturing complexity [1], n‐NaCl benefits from biocompatibility, simplicity, and regulatory familiarity, traits that favor rapid clinical testing [3, 4]. Local administration could be readily integrated into interventional oncology procedures, while systemic dosing may be optimized with pharmacokinetic modeling. Given the high prevalence of ionic imbalance across solid tumors, this strategy has broad applicability as an adjunct to checkpoint inhibitors or adoptive T‐cell therapy. Our results suggest that manipulating the tumor's ionic landscape using Cl^−^‐excess saline can bypass conventional immunosuppressive barriers by restoring the functionality of dendritic cells and T lymphocytes, two essential players in effective antitumor responses. This approach may be particularly relevant for tumors with poor immune infiltration, where ionic dysregulation is both a hallmark and a driver of immune exclusion. Of interest, our previous integrative analyses of TCGA datasets (not shown) revealed that tumors with high expression of Na⁺/K⁺‐ATPase subunits (e.g., ATP1A1, ATP1B1), potential mediators of ionic resilience, tended to exhibit low immune cytolytic activity but responded better to checkpoint inhibitors [5]. Such features may serve as potential biomarkers to stratify patients most likely to benefit from ionic reprogramming strategies.

In summary, we present Cl^−^‐excess n‐NaCl as a minimalist yet effective immune‐enhancing intervention that directly targets ionic stress in the tumor milieu. As an add‐on to existing immunotherapies, this formulation has the potential to enhance immune priming, amplify effector cell function, and convert “cold” tumors into immunologically active ones with minimal systemic burden. Supplementary Materials

## Author Contributions

G.W. and C.W. conceived the idea and designed the experiments. C.W., Y.D., H.L., A.W., S.Y., M.X., and C.L. performed the most experiments and analyzed data. J.F., Z.W., and G.W. provided administration and supervision. G.W. wrote the manuscript. All authors have read and approved the manuscript.

## Conflicts of Interest

The authors declare no conflict of interest.

## Ethics Statement

All animal experiments were conducted in accordance with institutional guidelines and approved by the Institutional Animal Care and Use Committee of Sun Yat‐sen University (Approval No. SYSU‐IACUC‐2020‐B1041).

## Supporting information




**Supporting File 1**: mco270534‐sup‐0001‐SuppMat.docx

## Data Availability

All data generated or analyzed during this study are included in this published article and available upon reasonable request.

## References

[1] R. Eil , S. K. Vodnala , D. Clever , et al., “Ionic Immune Suppression Within the Tumour Microenvironment Limits T Cell Effector Function,” Nature 537 (2016): 539–543. 27626381 10.1038/nature19364 PMC5204372

[2] S. Feske , E. Y. Skolnik , and M. Prakriya , “Ion Channels and Transporters in Lymphocyte Function and Immunity,” Nature Reviews Immunology 12 (2012): 532–547. 10.1038/nri3233 PMC3670817 22699833

[3] D. Soll , C. F. Chu , S. Sun , et al., “Sodium Chloride in the Tumor Microenvironment Enhances T Cell Metabolic Fitness and Cytotoxicity,” Nature Immunology 25 (2024): 1830–1844. 39198632 10.1038/s41590-024-01918-6 PMC11436365

[4] C. Scirgolea , R. Sottile , M. De Luca , et al., “NaCl Enhances CD8(+) T Cell Effector Functions in Cancer Immunotherapy,” Nature Immunology 25 (2024): 1845–1857. 39198631 10.1038/s41590-024-01923-9

[5] K. Yang , Z. Li , Y. Chen , et al., “Na, K‐ATPase alpha1 Cooperates With Its Endogenous Ligand to Reprogram Immune Microenvironment of Lung Carcinoma and Promotes Immune Escape,” Science Advances 9 (2023): eade5393. 36763655 10.1126/sciadv.ade5393 PMC9916986

