# Supplementary material for: Non‐stoichiometric Sodium Chloride Reprograms Ionic Homeostasis to Enhance Antitumor Immunity
Source: MedComm (2020). 2025 Nov 29;6(12):e70534. doi: 10.1002/mco2.70534 (PMC12664901; doi:10.1002/mco2.70534)
Supplement: Supplementary file 1 — Supporting File 1: mco270534‐sup‐0001‐SuppMat.docx [file MCO2-6-e70534-s001.docx]

Supplementary Methods for

**Non-stoichiometric sodium chloride reprograms ionic homeostasis to enhance antitumor immunity**

Chongxiao Wang^1,#,*^, Yuan Deng^1,#^, Heng Liang^1,#^, Arabella H. Wan^1,#^, Shijia Yan^1^, Min Xiao^1^, Chuwei Liu^1^, Juan Fang^2^, Zhi Wang^2,*^, Guohui Wan^1,*^

^1^ State Key Laboratory of Anti-Infective Drug Discovery and Development, National-Local Joint Engineering Laboratory of Druggability and New Drug Evaluation, Schools of Pharmaceutical Sciences & Materials Science and Engineering, Sun Yat-Sen University, Guangzhou 510006, China

^2^ Hospital of Stomatology, Guanghua School of Stomatology, Sun Yat-Sen University, Guangzhou 510055, China

^#^ These authors contributed equally.

* Corresponding author

*Correspondence

Guohui Wan, School of Pharmaceutical Sciences, Sun Yat-Sen University, Guangzhou, Guangdong 510006, China

E-mail: [wanguoh@mail.sysu.edu.cn](mailto:wanguoh@mail.sysu.edu.cn)

Chongxiao Wang, School of Pharmaceutical Sciences, Sun Yat-Sen University, Guangzhou, Guangdong 510006, China

E-mail: [wchongx@mail.sysu.edu.cn](mailto:wchongx@mail.sysu.edu.cn)

Zhi Wang, Guanghua School of Stomatology, Sun Yat-Sen University, Guangzhou, Guangdong 510055, China

E-mail: [wangzh75@mail.sysu.edu.cn](mailto:wangzh75@mail.sysu.edu.cn)

**Materials and Methods**

**Preparation of non-stoichiometric NaCl solutions**

NaCl (1 g) was dissolved in 300 g ultrapure water (tank C); tanks A and B contained 40 g ultrapure water. Electrochemical cells (10 cm² electrode area, 1 cm spacing) were operated at 1 V and 20 °C with three pumps (10 ml/min) for 4 h. Ion exchange membranes were placed 1 mm from electrodes. Samples from tanks A and B were analyzed by ion chromatography and concentrated by rotary evaporation (50 °C, 100 mbar) to yield stable n-NaCl solutions.

**Determination of chloride and sodium concentrations**

Ion chromatography (Dionex ICS-5000, Thermo Scientific) was used to measure Cl⁻ and Na⁺ contents in n-NaCl solutions. For anions, samples were analyzed using an IonPac AS22 analytical column (250 × 4 mm) with an AG22 guard column (50 × 4 mm), isocratic elution with 4.5 mmol/L Na₂CO₃ and 1.4 mmol/L NaHCO₃, flow rate 1.0 mL/min, and suppressor current 30 mA. For cations, an IonPac CS12A analytical column (250 × 4 mm) with a CG12A guard column (50 × 4 mm) was used, elution with 18 mmol/L methanesulfonic acid, flow rate 1.0 mL/min, and suppressor current 54 mA.

**Cell culture**

BMDCs and CD8⁺ T cells were isolated from BALB/c mice. MC38, CT26, Jurkat T, and DC2.4 cells (Procell, China) were cultured in RPMI 1640 or DMEM with 10% FBS and 1% penicillin/streptomycin at 37 °C in 5% CO₂. All lines were tested mycoplasma-free.

**Flow Cytometry analysis of intracellular Na^+^, K^+^ and Ca^2+^ content**

Intracellular Na^+^ was measured by Enhanced NaTrium Green-2 AM (ENG-2 AM) (MX4514, MKBio, China) according to the manufacturer’s protocols. MC38, Jurkat T and DC2.4 cells were respectively treated with 5 μM ENG-2 AM and Pluronic F-127 (MS4301, MKBio, China) for 30 min. After centrifugation (400×g for 5 min), cells were resuspended in the culture medium with or without 202 as indicated. The fluorescence intensity of ENG-2 AM were quantified and followed by flow cytometry analysis (Beckman, USA). Intracellular K^+^ was measured by Enhanced Potassium Green-4 AM (EPG-4) (MX4521, MKBio, China) according to the manufacturer’s protocols as Na^+^. Intracellular Ca^2+^ was measured by Fluo-4 (MX4504, MKBio, China) according to the manufacturer’s protocols as Na+.

**Co-incubation of tumor cells, bone marrow-derived DCs, and spleen lymphocytes**

BMDCs were generated from BALB/c or C57BL/6 mice by culturing bone marrow cells in GM-CSF (20 ng/ml, 6 days). On day 7, DCs were treated with #202 for 48 h, stained for MHC-II and analyzed by flow cytometry and RT-qPCR.

CD8⁺ T cells were isolated from spleens using density gradient centrifugation and magnetic sorting. DCs and CD8⁺ T cells were co-cultured with CT26 (BALB/c) or MC38 (C57BL/6) tumor cells pretreated with #202 for 24 h. After 48 h, lymphocytes were stained for CD8 and IFN-γ and analyzed by flow cytometry; supernatants were assayed for IL-2 and IFN-γ (ELISA, CUSABIO).

**Quantitative RT-PCR**

Total RNA was isolated using Trizol reagent according to the manufacturer’s instruction (ThermoFisher, USA) and cDNA was synthesized by using the PrimeScript RT reagent Kit (RR036A, Takara, Japan). The resulting cDNA was used for quantitative RT-PCR by using SYBR-Green Master mix (RR820B, Takara, Japan) in 7500 apparatus (Applied Biosystems).

**Quantification of cytokines by enzyme-linked immunosorbent assay (ELISA)**

The concentrations of cytokines were estimated for each experimental condition by ELISA, using commercial kits purchased from CUSABIO, according to the manufacturer’s instructions. The cytokine kits included IL-2 (CSB-E04627m), IFNγ (CSB-E04578m), TNFα (CSB-E04741m). Positive controls were supplied in the kit.

***In vivo* antitumor study**

Female BALB/c, C57BL/6, and BALB/c-nu mice (5–7 weeks old, Laboratory Animal Centre of Sun Yat-Sen University) were housed under SPF conditions. All procedures were approved by the Institutional Animal Care and Use Committee of Sun Yat-sen University.

For the MC38 model, 5 × 10⁵ cells were injected subcutaneously into the back of BALB/c mice. After 1 week, mice were randomized into four groups and treated intratumorally with water, #1, #201, or #202 once daily. Body weight and tumor volume (calculated as a² × b/2, with a = shortest diameter, b = longest diameter) were measured every 2 days. Rationale: group sizes were based on pilot experiments to ensure statistical power while minimizing animal use.

For efficacy studies, MC38, CT26, and Hepa1-6 tumor-bearing mice were assigned to eight groups and treated daily with water, #173, #175, #201, or #202, with or without anti–PD-1 antibody (BE0146, Bio X Cell; i.p. twice weekly). To evaluate effects in immunodeficient hosts, Hepa1-6 tumor-bearing BALB/c-nu mice were randomized into three groups (water, #201, #202). After 14 days, mice were sacrificed, blood collected for biomarker analysis, and organs (heart, liver, spleen, kidney, tumor) harvested for histology. Tumor inhibition rate (TIR) was calculated as [1 – (mean tumor weight of treated / control)] × 100 and reported in the Results.

**RNA-sequencing**

The mRNA sequencing program was performed at LC-Bio Technology Co., Ltd (Hangzhou, China). Briefly, the total RNAs were isolated from CT26 tumor samples using TRIzol agent (Invitrogen, CA, USA). After establishing sequencing libraries, samples were subjected to perform the 2 × 150 bp paired-end sequencing (PE150) on an Illumina Novaseq™ 6000.

**Statistical analysis**

Data were analyzed in GraphPad Prism 7.0. Student’s unpaired t-test was applied; results are mean ± SD. P < 0.05 was considered significant.
